# Distinctive Gut Microbiota of Honey Bees Assessed Using Deep Sampling from Individual Worker Bees

**DOI:** 10.1371/journal.pone.0036393

**Published:** 2012-04-27

**Authors:** Nancy A. Moran, Allison K. Hansen, J. Elijah Powell, Zakee L. Sabree

**Affiliations:** Department of Ecology and Evolutionary Biology, Yale University, New Haven, Connecticut, United States of America; Ghent University, Belgium

## Abstract

Surveys of 16S rDNA sequences from the honey bee, *Apis mellifera*, have revealed the presence of eight distinctive bacterial phylotypes in intestinal tracts of adult worker bees. Because previous studies have been limited to relatively few sequences from samples pooled from multiple hosts, the extent of variation in this microbiota among individuals within and between colonies and locations has been unclear. We surveyed the gut microbiota of 40 individual workers from two sites, Arizona and Maryland USA, sampling four colonies per site. Universal primers were used to amplify regions of 16S ribosomal RNA genes, and amplicons were sequenced using 454 pyrotag methods, enabling analysis of about 330,000 bacterial reads. Over 99% of these sequences belonged to clusters for which the first blastn hits in GenBank were members of the known bee phylotypes. Four phylotypes, one within Gammaproteobacteria (corresponding to “Candidatus Gilliamella apicola”) one within Betaproteobacteria (“Candidatus Snodgrassella alvi”), and two within *Lactobacillus*, were present in every bee, though their frequencies varied. The same typical bacterial phylotypes were present in all colonies and at both sites. Community profiles differed significantly among colonies and between sites, mostly due to the presence in some Arizona colonies of two species of Enterobacteriaceae not retrieved previously from bees. Analysis of Sanger sequences of rRNA of the Snodgrassella and Gilliamella phylotypes revealed that single bees contain numerous distinct strains of each phylotype. Strains showed some differentiation between localities, especially for the Snodgrassella phylotype.

## Introduction

The honey bee, *Apis mellifera*, is domesticated around the world for honey production and crop pollination. As the most important agricultural pollinator, it is a key link in the human food supply. Honey bees exhibit a highly advanced social system, in which most individuals are non-reproductive females (workers) that provision and rear young within large colonies. Since 2006, losses of honey bee colonies have brought attention to the need for understanding the microbial associations of this species, including both symbiotic and pathogenic interactions [1]–[3].

Several studies using different non-culture based sequencing methodologies have pointed to a distinctive set of bacteria present in the guts of healthy adult worker honey bees collected in Europe, North America, Australia, Africa and Asia [1], [4]–[8]. Retrieved sequences correspond to about 8 bacterial phylotypes, and some of these phylotypes also have been isolated from bumble bees (genus *Bombus*) in North America and Europe [8], [9]. All but one of these phylotypes appear to be restricted to species of *Apis* and *Bombus* and absent from non-social bees or non-bee environments [8]. Furthermore, over 95% of bacterial sequences retrieved from honey bees have belonged to these eight phylotypes [8]. A study of the establishment of three of these phylotypes during worker development showed that each has a characteristic pattern of colonization within the gut [10].

While studies to date document a set of phylotypes that are widespread in honey bees and not sampled outside bees, results so far do not give a clear picture of the constancy of the honey bee gut community among individual bees, colonies, and geographic locations. No study of gut microbiota in honey bees has addressed variation among individual bees, and surveys have retrieved only limited numbers of sequences per sample. Of the two studies with the most extensive sampling, one included 538 sequences from two Arizona samples [8], and the other included 496 sequences from several pooled samples representing healthy and diseased colonies [1]. In particular, whether each of the eight phylotypes is present in every worker bee is not evident from previous data, as most studies have relied on pooled samples from several bees. Furthermore, rare phylotypes are expected to be missed by most studies to date, given the limited depth of sequencing.

In this study, we report results from deep sampling of bacterial gut communities of individual honey bees, using 454 pyrotags for diagnostic regions amplified from the 16S rRNA gene, a method originally applied to marine bacterial diversity [11]. We compare gut communities for different worker bees within colonies, for different colonies at the same site, and for two North American sites, Arizona and Maryland, that are both geographically and environmentally divergent. For two of the phylotypes, corresponding to recently proposed “*Candidatus* Snodgrassella alvi” and “*Candidatus* Gilliamella apicola” (hereafter referred to as “Snodgrassella” and “Gilliamella” phylotypes), longer sequences of 16S rRNA were acquired to examine the extent of strain variation within individuals bees.

## Methods

### Bee samples and preparation

Each sample consisted of genomic DNA extracted from the gut of a single worker bee taken from the outer frames within colonies. Based on studies of the relationship between worker age and behavioral traits [12], bees in this location are expected to be guard bees, of ∼16 days of age although other workers might occasionally be included; a study of colonization of the worker gut suggested that colonization occurs by day 9 following emergence from the pupal stage [10]. We sampled 2 localities, consisting of the USDA Agricultural Research Service Bee Labs in Tucson, Arizona and in Beltsville, Maryland, on 4/28/2011 and 4/20/2011 respectively. In each location, 5 bees were sampled from each of 4 colonies, for a total of 40 samples representing individual bees.

Bees were preserved in 95% ethanol after collection and prior to dissection. Whole guts from ventriculus to rectum were aseptically dissected from 5 randomly selected workers for each colony. The dissected guts were placed in a sterile 1.5 mL pestle tube with 710 µl buffer AG (200 mM NaCl, 200 mM Tris, 20 mM EDTA, plus 6% SDS) and were homogenized by maceration with scissors and then crushed with a disposable sterile pestle (Bel-Art Products). The homogenate was then added to a sterile bead tube containing 500 µl of phenol/chloroform/isoamyl pH 7.9 (Ambion) along with ∼500 µl 0.1 mm silica zirconia beads (BioSpec Products, Bartlesville, OK). The bead tubes were placed in a BioSpec high speed bead beater, beaten at the maximum setting for 3 min, then spun at 1000 RPM for 2 min. The resulting aqueous phase was extracted with a second phenol/chloroform/isoamyl preparation in a Light Phase Lock Gel tube (5 Prime). The aqueous phase of this extraction was collected and combined with 1/10 volumes sodium acetate pH 5.5 (American Bioanalytical) and an equal volume of isopropyl alcohol (American Bioanalytical). The samples were then allowed to incubate at −20°C overnight and then spun at 14,000 RPM for 30 min in a 4°C microcentrifuge. The pellets were washed with 70% ethanol and dried for 5 min in an unheated vacuum evaporator. The pellets were resuspended in 100 µl TE pH 8 (10 mM Tris pH 8 and 1 mM EDTA) and incubated for 30 min at 37°C with 2 µl RNAse A (Qiagen). These extracts were then further purified with a Qiagen QIAquick column and eluted in 30 uL Buffer EB (Qiagen). The final extracts were quantified using a Qubit dsDNA broad range assay (Invitrogen) and the resulting DNA samples were sent to the Joint Genome Institute (JGI).

### PCR and pyrosequencing

At JGI, the V6–V8 regions of the 16S rRNA gene of the samples were amplified in triplicate using universal 16S rRNA primers adapted with 454 FLX Titanium sequences. The forward primer was 926F454 Tit F 5′- CCTATCCCCTGTGTGCCTTGGCAGTCTCAG-aaactYaaaKgaattgacgg-3′ (Lib B adapter is in caps) and the reverse barcoded primer was 1392R454 Tit R 5′- CCATCTCATCCCTGCGTGTCTCCGACTCAG-NNNNN-acgggcggtgtgtRc (Lib A is in caps and the variable barcode region is denoted by N's). Amplicons were sequenced using Roche 454 Titanium sequencing (for details see http://www.jgi.doe.gov/sequencing/protocols/index.html).

### Pyrotag analysis

The retrieved sequences were processed by excluding sequences not matching the primers, with low quality for 10% or more of the sequence, or with fewer than 200 base pairs. These reads were deposited in the GenBank Sequence Read Archive (accession SRA046735). For analyses, all reads were trimmed to the same aligned 180 bp region. Retained sequences were then grouped into clusters of 100% identity and also into clusters of 97% or greater identity, using the “PyroTagger” pipeline [13]. These were classified into major taxonomic lineages using blastn against greengenes (greengenes.lbl.gov/) for prokaryotic sequences and against silva (www.arb-silva.de/) for eukaryotic sequences.

Our primers amplified rRNA from some eukaryotes, and the clusters included many bee sequences (19% of total), some plant or chloroplast sequences presumably from ingested pollen or nectar (0.2% of total), and a few fungal, and microsporidian sequences (totaling fewer than 0.1% of total), in addition to bacterial sequences (81% of total); no archaeal sequences were retrieved. Since we were interested in bacterial diversity, eukaryotic (including chloroplast) clusters were removed. Clusters that represented fewer than 1% of bacterial sequences in every sample were removed; most were present as one or few sequences in a single individual and absent from most samples. Only 11 clusters remained, and these represented 98.5% of all bacterial sequences in the dataset. These clusters were considered as Operational Taxonomic Units (OTUs) in our analysis. The top blastn hits against the GenBank nucleotide database were determined for representative sequences of each of these 11 OTUs. The gut community for each bee was represented as the proportion of sequences derived from each of the 11 OTUs.

To determine the sources of small clusters other than these 11, we used representative sequences from every bacterial cluster containing more than 3 sequences as queries in blastn searches against GenBank. Sequences with the same first hits were pooled, yielding only 10 groups. These 10 groups corresponded to the same 11 clusters as in the first analysis (with two pooled together) but also included small clusters closely related to the large clusters. These 10 clusters included 99.98% of the bacterial sequences.

For richness and evenness estimates, EstimateS [14] was used. Individuals were treated as sample units for each colony (N = 5 per colony). For richness estimates, ACE (abundance-based coverage estimator) [15] estimates were computed. Evenness of bacterial OTUs within each colony was estimated using Simpson's measure of evenness (E^1/D^), which is the reciprocal form of Simpson's dominance index (D) [16] divided by the number of species in the sample [17]. An ANOVA was used to test mean differences of OTU evenness between AZ and MD sites [17]. Non-parametric Kolmogorov-Smirnov Z was used to test mean differences of OTU richness between AZ and MD sites [18].

To explore differences among individuals, colonies and sites, we used multivariate analyses of community profiles. OTUs were defined as the 11 sequence clusters comprising 98.5% of the bacterial sequences. In a second analysis, OTUs were defined as the 10 clusters corresponding to diagnostic blastn hits (above) and containing 99.98% of the sequences. The two approaches gave very similar results, and only the first is reported.

Bacterial sequence reads per individual bee were standardized to the same sample size before multivariate community analyses were conducted. Standardization was carried out by randomly selecting 948 reads (the smallest sample size) per individual using Perl.

For all community multivariate analyses, PCORD (version 4.25) was used [19]. Multi-Response Permutation Procedures MRPP [20] were used to test for differences among *a priori* groups (bee colonies and sites). Nonmetric multidimensional scaling (NMS) [21], [22] was used to compare bee gut microbiota assemblages among bee individuals. One extreme outlier (bee individual AZ_109_3) was removed, since extreme outliers distort ordination solutions. All criteria, distance measures, and parameters chosen are similar to those in Hansen et al. [23]. Briefly, a two-dimensional solution was chosen based on a combination of low stress, final instability, and P-values. 500 iterations were conducted. Non-parametric indicator species analysis (ISA) [24] was used to identify the OTUs that best described differences between sites, based on two independent measurements of species distribution, specificity and fidelity (i.e., an OTU was specific to a particular group (specificity) and widespread in all samples of that group (fidelity)). Potential indicator values (IndVal) that can result from ISA range from 0–100 where values >25 signify a good indicator [24]. Parameters and criteria are similar to those in Hansen et al. [23].

### Strain variation within Snodgrassella and Gilliamella

Because pyrotag sequences were too short for fine-scale discrimination of strains within each phylotype, we obtained Sanger sequence data to assess the extent of strain variation within the Snodgrassella and Gilliamella phylotypes. Reverse primers were designed to complement sequences within the region used to align pyrosequence reads (positions between 1100 to 1300). The Snodgrassella primer (betacd1 5′- TTCGCTACCCTCTGTACCGACCATT - 3′) or Gilliamella primer (gma1cd2 5′-TCGCCTCCCTTTGTATACGCCATT-3′) were paired with 16S rRNA universal primer 27Fshort (5′-GAGTTTGATCCTGGCTCA-3′) in 25 µL reactions of 0.8 U Taq DNA Polymerase (New England BioLabs), 0.25 mM of each dNTP, 1x PCR buffer, and 0.5 µM concentrations of each primer. Fifty ng of DNA from individual bee samples: AZ100.3, AZ107.2, AZ109.2, AZ125.5, MD19.5, MD216.5, MD299.4 and MD365.2 were added to these reactions, and nuclease-free water was used in the negative control. These targeted regions of 16S rRNA sequence were amplified via PCR as follows: 4 min at 94°C; 35×30 s at 94°C; 30 s at 50°C, 1 min at 72°C; 10 min at 72°C. PCR products were visualized on a 1% agarose gel run at 90 V for 40 min and stained with ethidium bromide. Amplified PCR product was cleaned using Agencourt Ampure XP beads (Beckman Coulter), ligated into pGEM-T vector (Promega) and used to transform chemically competent DH5α *E. coli* (Invitrogen) using the manufacturer's recommended protocol. Twenty-four transformants were selected from each library, and inserts were verified by colony PCR using T7 (5′-TAATACGACTCACTATAGGG-3′) and SP6 (5′-ATTTAGGTGACACTATAG-3′) primers. The following amplification protocol was used: 1×94°C for 2 min; 28×10 s at 94°C, 20 s at 46°C for 20 s, 90 s at 72°C; 1×72°C for 1 min. Three µl of this reaction mix was run on an agarose gel and visualized to ensure incorporation of the approximately 1300 bp insert. DNA was then cleaned with Ampure beads and sent for bidirectional Sanger sequencing at the Yale Science Hill DNA Facility using T7 or SP6 sequencing primers.

We checked returned sequences using the RDP Classifier v2.2 [25], and discarded a single sequence from the Snodgrassella set not matching Betaproteobacteria. The remaining sequences were curated using Geneious, version 5.5 [26]. They were trimmed of vector sequence and checked for quality. Poor quality reads (<800 bp) were discarded (1 from the Snodgrassella set and 7 from the Gilliamella set). Sequences were checked for chimeras using the Greengenes Bellerpheron tool (greengenes.lbl.gov/), resulting in elimination of three additional sequences from the Snodgrassella set. Remaining sequences were aligned for each set separately, using MUSCLE within Geneious with a maximum of 8 iterations. Equivocal base calls and gaps were inspected and corrected as necessary. Duplicate sequences were removed, and one of each was used for the alignment (see Table 1) All of the nearly full-length sequences were aligned along with representative sequences from bacteria from other studies of *A. mellifera* and of related host taxa (e.g. *Bombus spp.*), and divergent sequences were used as queries in blastn searches of GenBank nucleotide data [27] to ensure that they had a strong match to the respective relevant phylotype. Three Gammaproteobacterial sequences from bee AZ109.2 had highest matches to *Serratia* species and were removed from the Gilliamella alignment. Blastn searches and neighbor-joining trees for the Gilliamella phylotype showed that a subset of sequences was derived from the related Gamma2 phylotype; these were excluded from analyses of strain variation. Alignments were edited and parsimony uninformative sites removed using MEGA [28]. Strain variation was assessed using DNAsp package [29] to estimate average pairwise sequence divergence, minimum number of recombination events, and polymorphisms restricted to a single locality or shared between localities. In the case of Gilliamella, a large number of recombination events was evident. A phylogenetic tree based on sequence data assumes clonal or near-clonal replication of the sequence and no recombination between sequences; the relationships of Gilliamella sequences would be better represented by a complex web than a tree. Therefore, no phylogenetic tree was constructed. In the case of Snodgrassella, a Neighbor-Joining tree was built using a Tamura-Nei distance model, within MEGA [28]. Representative database sequences from the Snodgrassella phylotype were included in the analysis. Sequences derived from *Bombus* formed a more divergent cluster, and these were used as an outgroup to the *A. mellifera*-derived sequences.

**Table 1:** Summary of Pyrotag Reads from Honey Bee Samples, Including the Representation of Known Bee Phylotypes.

**All sequences:**	
# raw reads retrieved	530583
# reads matching primers	477986
# reads passing quality control	409086
Average read length	436 bp
# 100% id clusters	40879
# 97% id clusters	397
# honey bee 18S rRNA reads	78,248 (19.1% of total)
# Bacterial 16S rRNA reads	329,550 (80.6% of total)
**Bacterial sequences only:**	
Average # bacterial sequences per bee	8239 (range 957–11873)
# 97% id clusters	336
# 97% clusters contributing >1% of reads in any sample	11
# reads in these 11 clusters	324,492
% total bacterial reads in these 11 clusters	98.47%
% bacterial sequences in known bee phylotypes (99.10%):	
Firm5	45.44%
Firm4	23.18%
Gilliamella (Gamma1)	11.92%
Snodgrassella (Beta)	9.14%
Bifido	5.41%
Gamma2	1.98%
Alpha2	1.02%
Alpha1	0.97%
% bacterial sequences in phylotypes novel in bees (0.9%)	
Gamma3	0.64%
Gamma4	0.29%

Final sequences for these phylotypes were deposited in GenBank with the accession numbers JQ581680-JQ582008

### Characterization of 16S rRNA sequences for Gamma3 and Gamma4 phylotypes

To further characterize the novel clusters (Gamma3 and Gamma4), ∼50 ng of DNA from individual bee preps (AZ107.4, AZ109.1, and AZ109.3) was used to perform PCR amplification of near full-length 16S rRNA sequences, using the following universal primers: 27F′-HT (5′-AGRGTTTGATYMTGGCTCAG-3′) and 1492R′-HT (5′-GGYTACCTTGTTACGACTT-3′) [30]. PCR product was cleaned using Agencourt Ampure XP beads (Beckman Coulter), ligated into pGEM-T vector (Promega) and used to transform chemically competent DH5a *E. coli* (Invitrogen) using the manufacturer's recommended protocol. Transformants were selected from each individual bee's library, and inserts were screened by colony PCR using the T7 primer and Gamma3/Gamma4 specific primers. Lack of primer specificity resulted in no suitable sequences for Gamma3. Gamma4 was targeted by the reverse primer γ4R1 (5′- GTGCTACAATGGCGCATACA -3′) using a PCR protocol with initial denaturation of 94°C for 4 min followed by a 11 touchdown cycles of 30 s denaturation at 94°C, 20 s annealing step declining from 57°C to 46°C, and 1.5 min elongation step at 72°C, then 30 standard cycles with annealing at 46°C and final extension at 72°C for 5 min. Resultant positive amplifications were Sanger-sequenced at the Yale, Science Hill DNA Facility and aligned to the Gamma4 reads from the pyrosequencing dataset. Three nearly full length sequences showing identity or near identity (∼100%) to the Gamma4 reads were deposited in GenBank under the accession numbers JQ582009-JQ582011. Blastn of these Gamma4 sequences reveals similarity (≤96%) to members of the family Enterobacteriaceae.

## Results

### Description of phylotypes retrieved from bee guts

Following quality control, a total of 329,550 bacterial sequences were retrieved, and these formed 336 clusters of 97% or greater sequence identity (Table 1). Of these, only 11 clusters constituted at least 1% of sequences in any single bee. In nine of these 11 clusters, the top blastn hits corresponded to sequences for characteristic phylotypes previously sampled from bees. Seven typical bee phylotypes were represented by a single one of these clusters (“Alpha1”, “Alpha2”, “Gilliamella”, “Gamma2”, “Snodgrassella”, “Firm5”, and “Bifido”), and one typical bee phylotype (“Firm4”) was represented by two clusters (using the same phylotype names as in Cox-Foster et al. [1] and Martinson et al. [8], [10]). The other two clusters corresponded to distinct Gammaproteobacteria not previously reported from bees, and were given the names “Gamma3” and “Gamma4”. These 11 clusters made up 98.5% of the total bacterial sequences in the dataset and were considered as OTUs in analyses of community structure.

To explore the identity and source of the smaller clusters, we used blastn of representative sequences to determine whether they also represented characteristic bee phylotypes, or whether they represent diverse bacteria from other lineages. Most of the smaller clusters had top hits corresponding to a characteristic bee phylotype. Clusters with top hits to the 8 characteristic bee phylotypes constituted 99.10% of all bacterial sequences retrieved, and Gamma3 and Gamma4 sequences comprised another 0.8%. Thus, in our total dataset, more than 99% of bacterial rRNA sequences belonged to the previously recognized bee-associated phylotypes.

**Figure 1:**
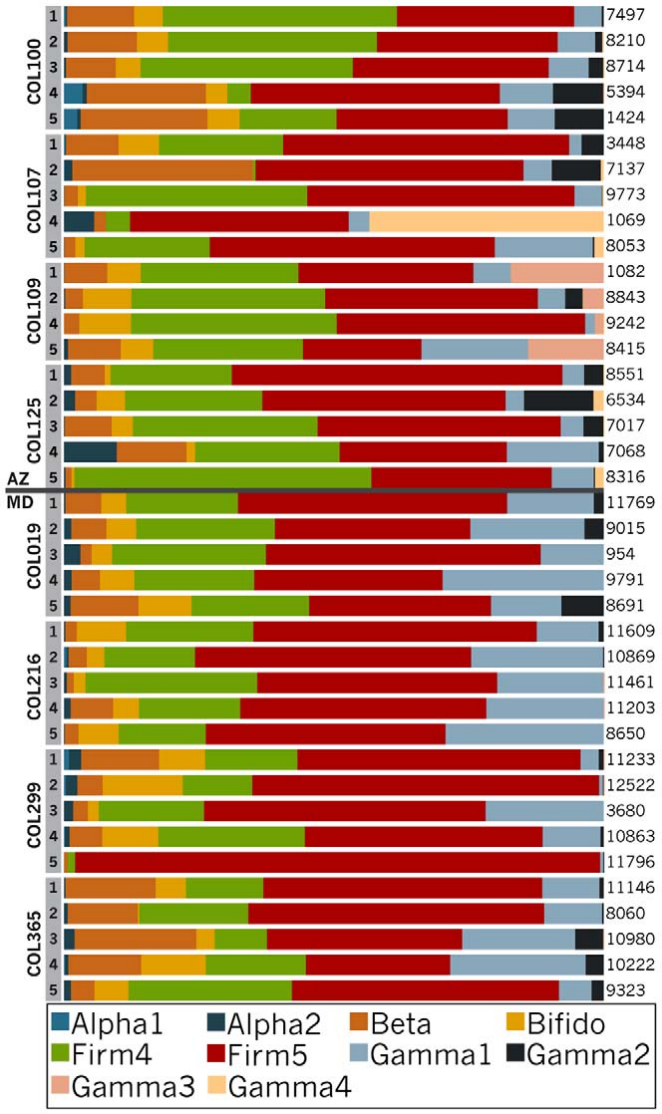
Bar graph showing relative abundances of bacterial phylotypes within the guts of individual honey bees from colonies in Arizona and Maryland.

Qualitative assessment of the distribution of the phylotypes revealed that every bee has a large proportion of the two Lactobacillus phylotypes, termed Firm4 and Firm5 (Fig. 1). Together, these comprised the majority of sequences in most (34/40) samples, ranging from 20% to 99% of sequences per sample. Furthermore, every bee contained the Gilliamella phylotype, which comprised 0.6–30% of each sample, as well as the Snodgrassella phylotype, which ranged from 0.6–39% of each sample. Most bees contained the Alpha2 phylotype (36/40), the Bifido phylotype (37/40) and the Gamma2 (33/40), each at low frequencies. The Alpha1 was erratically present, occurring in 14/40 bees and usually at low frequency when present.

Gamma3 and Gamma4, the 2 OTUs present at 1% or more in at least one sample and not matching sequences previously sampled from bees, both fell within Enterobacteriaceae. Gamma3 was present in all individuals of a single colony from AZ (Colony 109) and sporadically in other individuals from AZ and MD. Gamma4 was present in 4 of 5 individuals from a single colony from AZ (colony 107) and at very low frequency in several other AZ bees; it was absent from all MD bees.

### Variation among individuals, colonies, and sites in relative abundances of the characteristic phylotypes

We considered the set of 11 OTUs, accounting for 98.5% of bacterial reads, in analyses to determine whether gut community profiles vary among individuals, colonies, or sites. Comparing the AZ and MD sites, all 11 of the OTUs were found at the AZ site, and 10 were found in MD (all but Gamma4). Average OTU richness for individual bees at the two sites were 9.75 and 9, respectively, and not significantly different (Z = 1.061, P = 0.211, Table 2). The other metric of diversity, evenness, was not significantly different between AZ and MD sites (F = 1.55, P = 0.259; Table 2).

**Table 2:** Diversity Metrics and Compositional Variation of Bee Gut Bacteria Among Colonies and Between Sites.

	Estimated species richness	Evenness	MRPP Colony	MRPP Site	Beta diversity
Colony name	ACE(SD)	E^1/D^ (SD)	Average within group distance	Average within group distance	Average half change^1^
AZ100	10(0)	0.4290(0)	0.227		
AZ107	10(0)	0.3340(0)	0.389		
AZ109	10(0)	0.5130(0)	0.408		
AZ125	9(0)	0.3733(0)	0.272	0.345	0.6107
MD019	9(0)	0.3733(0)	0.193		
MD216	9(0)	0.3533(0)	0.144		
MD299	9(0)	0.2611(0)	0.314		
MD365	9(0)	0.4111(0)	0.266	0.249	0.413

1 Average half-change = log (1-average within group distance)/log (0.5), (half change = amount of compositional and structural change resulting in 50% dissimilarity among colonies (34)).

**Figure 2:**
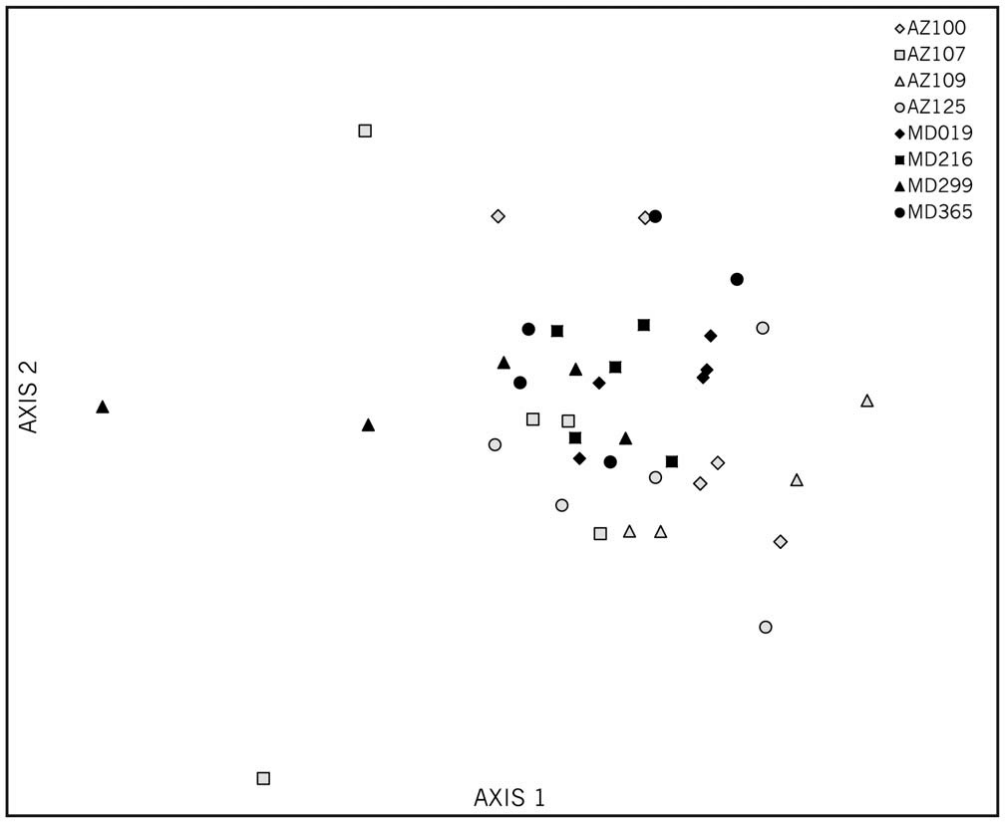
Nonmetric multidimensional scaling ordination of gut bacterial assemblages from individual honey bees (N = 39). (2D solution, 500 iterations, final stress = 17.56, final instability = 0.0008, Monte Carlo P = 0.0099, 0.0396). Axis 1 explains 65.5% of the variation, and Axis 2 explains 23.3% of the variation in the gut communities. Individuals from the same colony are represented by the same shape and color.

Thus, aside from the Gamma3 and Gamma4 OTUs, which do not correspond to known bee phylotypes, the representation of OTUs is the same for the two sites. All or most individual bees contain 7 of the 8 typical bee phylotypes (all except Alpha1). However, as noted, frequencies of each OTU vary considerably. To determine whether assemblages differ between colonies and sites, multivariate analyses, NMS and MRPP, were used. Bacterial community profiles were significantly different between AZ and MD sites (MRPP: *T* = −5.465, *A* = 0.048, *P* = 0.0004). NMS ordination results reflect this difference as bee individuals tend to cluster by site (Fig. 2). AZ colonies are more heterogeneous in bacterial community profiles based on a higher MRPP within-group dissimilarity distance (*D*) and beta diversity (Table 2); this is reflected in closer clustering of the MD individuals in the NMS ordination (Fig. 2). Indicator species analysis showed Gamma3 and Gamma4 (the novel phylotypes) associated with the AZ samples and Firm5 and Gilliamella associated with MD samples (Table 3). (In other words, although Firm5 and Gilliamella were present in every bee they were relatively abundant in the MD individuals.) On a colony level, bacterial OTU community composition and structure were significantly different across the 8 bee colonies (MRPP: *T* = −4.54, *A* = 0.114, *P* = 0.0001). Within-group dissimilarity distances (*D*) were highly variable among colonies (Ave = 0.276, SD = 0.09), particularly among AZ colonies (Table 2).

**Table 3:** Indicator Species Analysis Identifying OTUs that Characterize Arizona and Maryland Sites.

Site	Indicator OTU	Indicator value	P-value^1^
MD	Firm 5	54.8	0.04
	Gilliamella	67.8	0.001
AZ	Gamma 3	35	0.011
	Gamma 4	40	0.002

1 P-value is based on Monte Carlo test with 1,000 permutations.

The phylotypes corresponding to Gamma3, Gamma4, and Alpha1 had the most variable occurrence across individuals. Both Gamma3 and Gamma4 were largely confined to particular AZ colonies (colonies 107 and 109). Alpha1, which has been previously retrieved from honey bees, has an erratic distribution across individuals. Potentially, high frequencies of these three organisms correlate with disruption of the normal gut microbiota. An analysis of the effect of these 3 OTUs on the profile of the remaining microbiota shows significant associations with relative abundances of other bee phylotypes (Table 4).

**Table 4:** Potential effects of sporadic OTUs on bee gut core communities among individuals (N = 40) based on Indicator Species Analysis.

Sporadic OTUs	Indicator OTUs	Association^1^	INDV	P
Alpha1				
	2 Firm4	−	59.9	0.029
	4 Snodgrassella	+	70.6	0.008
	5 Bifido	+	61.3	0.03
Gamma3				
	1 Firm5	−	57.9	0.013
Gamma4				
	3 Gilliamella	−	65.7	0.023
	5 Bifido	−	75.9	0.002

1 − indicates that the indicator OTU is more successful in hosts not harboring the sporadic OTU, whereas + indicates the opposite.

### Strain variation of phylotypes within and between colonies and sites

**Figure 3:**
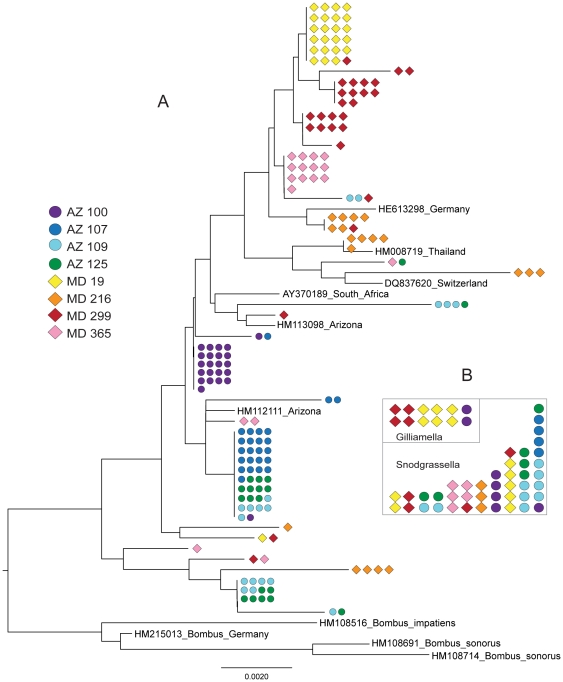
A. Phylogenetic tree based on amplified and cloned sequences of 16S rRNA genes for the Snodgrassella phylotype from individual bees collected at the Arizona and Maryland sites, and with several previously published sequences from bee-associated bacteria corresponding to the Snodgrassella phylotype. Singletons were removed before analysis so clusters at branch tips represent sequences that are identical or differ only in single sequences. **B**. Sets of identical sequences within the 1250 bp 16S rRNA sequences for Gilliamella and Snodgrassella. Each set of identical sequences is represented as a single column of symbols corresponding to colony and site of the sample. Identical sequences were only found for samples from the same site and were usually clustered within an individual bee.

Because the pyrotag sequences are too short for meaningful analysis of the strain variation within the phylotypes, we obtained longer sequences for a limited set of bees, for both the Snodgrassella and the Gilliamella phylotypes, using targeted PCR and Sanger sequencing. Following quality filtering, we retrieved 185 and 154 high quality sequences for the Snodgrassella and Gilliamella phylotypes respectively. For both, we observed strain variation, with average pairwise sequence divergence (π) estimated at 0.007 and 0.016 respectively (Table 5). Because sequences were obtained singly for a clone, singleton positions potentially reflect errors introduced during amplification, cloning or sequencing, or single base differences confined to one rRNA operon copy, potentially leading to overestimation of pairwise sequence divergence. However, several indicators show that much of the variation is genuine. First, many polymorphisms were not singletons but were present in multiple haplotypes. Second, the observed variation far exceeded that expected from error (usually less than 0.001%) [31], [32]. Third, sequencing errors could not give rise to the clustering of near-identical sequences within colonies and localities for the Snodgrassella phylotype (Fig. 3A) nor to the clustering of identical sequences within individual bees (or colonies) and within localities for both phylotypes (Fig. 3B). Fourth, some patterns were consistent across samples. For example, the Gilliamella phylotype had higher polymorphism than the Snodgrassella phylotype in each of the 8 samples (Table 5).

**Table 5:** Polymorphism analyses for cloned 16S rRNA sequences from the Snodgrassella and Gilliamella phylotypes.

	Snodgrassella			Gilliamella		
	Total	AZ	MD	Total	AZ	MD
# sequences	185	90	95	154	72	82
π- all data	0.0068	0.0052	0.0068	0.0160	0.0176	0.0131
Analyses excluding singletons (parsimony informative sites only):						
# polymorphic sites	49	33	42	130	110	82
Fixed differences between localities	0			0		
Polymorphic sites monomorphic in other locality		12	22		32	22
Proportion polymorphic sites confined to one locality	0.69			0.42		
Minimum # Recombination Events*	8			28		

* assuming no recurrent mutation.

For both phylotypes, every individual bee contained multiple sequence types. Both phylotypes also showed evidence of sequence recombination among strains, based on recombination tests across polymorphic sites (assuming no recurrent mutation). Snodgrassella showed a lower overall polymorphism level, fewer phylogenetically informative polymorphic sites (non-singletons), fewer recombination events, and more cases of identical sequences, as compared to Gilliamella (Table 5, Fig. 3B). Snodgrassella also showed more polymorphic sites restricted to one locality, with 69% of non-singleton polymorphisms limited to one location versus only 42% for Gilliamella. These results suggest that Snodgrassella shows more between-locality divergence than does Gilliamella.

A phylogenetic tree constructed for the Snodgrassella sequences revealed considerable clustering of sequences from the same individual as well as clustering within each of the two localities (Fig. 3A). Only a single bee was sampled per colony, so this clustering could represent colony-level differences. Variation among operons within genomes could be ruled out as a primary source of the phylogenetic signal, because similar sequences tended to cluster according to site. Several database sequences from other locations were included in the phylogenetic analysis. One sequence collected 3 years previously at the same AZ site fell within a cluster dominated by AZ sequences, whereas a second fell into a mixed cluster (Fig. 3A). Sequences from *A. mellifera* collected in Thailand, South Africa, Switzerland and Germany all fell within the MD clusters, whereas sequences from several collections of *Bombus* in North America and Europe formed a clade somewhat distinct from all *A. mellifera*-derived sequences. These results support the conclusion that *A. mellifera* Snodgrassella strains are divergent from those in *Bombus*, that specific locations may contain a somewhat distinctive profile of strains, as in the case of the AZ strains, but that there is considerable global mixing of strains, indicated by the clustering of strains from Europe and Asia with strains from MD.

For Gilliamella, the mean pairwise sequence divergence was higher than in Snodgrassella (Table 5), and very few identical sequences were retrieved (Fig. 3B). As in Snodgrassella, identical sequences only occurred within a site. A large number of recombination events were evident in the Gilliamella sequences, implying that relationships of these sequences do not take the form of a phylogenetic tree and would be better presented as a complex network. Therefore, no phylogenetic tree was constructed for Gilliamella.

## Discussion

A distinctive set of phylotypes is consistently present in individual bees and, on average, contributes more than 99% of the 16S rRNA sequences present in the gut of each bee. However, the relative frequencies of these phylotypes varies considerably even from bees sampled on the same day from a single colony.

Previous non-culture-based studies of bee gut microbiota sometimes failed to sample particular phylotypes from individual samples, but the limited sampling conducted in previous studies would be expected to miss low frequency phylotypes. Our results show that the Gilliamella (Gamma1), Snodgrassella (Beta), Firm4 and Firm5 are present in every bee and that Bifido, Gamma2 and Alpha2 are present in most bees (or possibly all, if frequencies are sometimes very low and thus sometimes missed even by our sampling). The Snodgrassella and Gilliamella phylotypes are also found in other *Apis* species and in *Bombus* species from different locations and environments [8], [9], raising the possibility that these bacterial lineages have coevolved with their hosts during the diversification of these bees.

The variation in phylotype profile could reflect age, short-term differences in physiology, or variation in health status of individuals. For example, the community profile before and after defecation would differ, if the hindgut has a non-random set of phylotypes. A previous study [1] sampled the same phylotypes but resulted in different abundance profiles, with Gilliamella the most frequent. This difference is likely to reflect the DNA extraction method used in the study. The bead-beating method that we used, or a long lysozyme digestion [8], appears to be more effective in releasing the DNA from Gram-positive organisms such as the *Lactobacillus* and *Bifidobacterium* species (Firm4 and Firm5). Also different PCR primers have been used in some studies, possibly favoring amplification of different phylotypes. Thus, abundance profiles cannot be compared across studies using different protocols.

The honey bee gut microbiota appears to be relatively simple and consistent across individuals, as compared to the gut microbiota of other insects, based on available studies that used non-culture based methods. For example, gypsy moth (Lepidoptera) larvae have a gut community that is highly dependent on diet [33]. *Drosophila melanogaster* individuals (Diptera) have highly variable gut communities composed of bacterial phylotypes that are the same or closely related to bacteria living in soil or other environments [34], [35]. Scarab beetle larvae also display large intraspecific differences in gut microbiota [36]. Termite species resemble honey bees in being eusocial and in possessing a highly distinctive set of gut microbes that is transferred through social interactions and that plays a central role in digestion of lignocellulytic components of the diet [37]. In the termites, however, the gut community is far more complex, with hundreds of constituent bacterial phylotypes [38], [39].

Several of the honey bee-associated phylotypes are closely related to bacteria found in guts of other insects. For example, the Alpha2 phylotype, which appears to include at least two distinct species [8], is close to acetic acid bacteria, such as *Acetobacter* and *Gluconacetobacter* species, and to phylotypes found in numerous insects, including other bees, mosquitoes, flies, leafhoppers and mealybugs [8], [40]. The Alpha1 phylotype, a relative of *Bartonella*, is closely related to phylotypes retrieved from numerous ant species [8]. The most consistently present proteobacterial phylotypes in honey bees, the Gilliamella, Gamma2, and Snodgrassella phylotypes, are highly distinctive and so far found only in honey bees and bumble bees [8], but the Glliamella and Gamma2 phylotypes are nested within a larger clade that has been recovered from guts of other insects [10], [41].

Most smaller pyrotag clusters display the same top blastn hits as large clusters. This potentially results from strain variation, divergence among 16S rRNA copies within a genome, or sequencing error that causes divergence from the main cluster [42]. To examine the possible impact of sequencing error in the pyrotag dataset, we considered the extent of error in the sequences corresponding to the 18S rRNA sequence of the honey bee, which is expected to show no variation as only one bee genotype was present. A large cluster contained 78,248 sequences with 100% match to the 18S rRNA of the honey bee, and 12 additional small clusters of similar sequences also had top blastn hits to honey bee (or other identical hymenopteran) sequences. These small clusters contained only 77 sequences in total, or 0.1% of the sequences with top blast hits to honey bee; they must result from sequencing error. This suggests that the small bacterial clusters also reflect sequencing error, although strain variation or divergence between operons within genomes may also contribute (as in the case of the larger clusters for Firm4).

The pyrotag data, consisting of short sequence tags from 16S rRNA, are not sufficient to assess strain variation within phylotypes. Previous studies that retrieved longer rRNA sequences did reveal some strain variation but included very few samples or sequences [8]. Our Sanger sequences for strains of the Snodgrassella and the Gilliamella phylotypes provide a larger dataset for assessing strain variation within and among individuals and localities (Table 5). We found clear evidence of strain variation within individual bees. Strains of the Snodgrassella phylotype show some clustering by location (Fig. 3). The founding of colonies by large numbers of individuals and the frequent exchange of bacteria by trophallaxis or other social interactions would be expected to facilitate the maintenance of strain variation within colonies and individuals, by preventing inoculation bottlenecks. Since rRNA is highly conserved, the observed variation is expected to correspond to greater differences at the level of whole genomes. Addressing the issue of the extent of strain variation, and its implications for bee biology, will require analysis of other genomic regions.

The consistent presence of the same phylotypes in individual bees, and their presence in honey bees worldwide [1], [4]–[8] supports the hypothesis that these bacteria have central functions in bees. If so, variation in gut bacteria, including possible functional differences among strains within a phylotype, may be an important factor in honey bee biology and colony health, just as variation in gut microbiota has been implicated in the health of humans and other animals [43].
